# Supplementary material for: Infectious diseases burden and antibiotic prescribing patterns among primary care patients in Harare, Zimbabwe – a cross-sectional analysis
Source: PLOS Glob Public Health. 2025 Apr 8;5(4):e0004442. doi: 10.1371/journal.pgph.0004442 (PMC11977971; doi:10.1371/journal.pgph.0004442)
Supplement: S1 File — Number of presentations according to year and clinic.Table B Other diagnoses (includes all patients irrespective of hospital referral who did not receive any other diagnosis). Table C Characteristics of patients according to recorded temperature. Table D Antibiotic prescribing according to month of presentation. Fig A Map of Harare representing the clinics included in the study. Fig B Comparison of population age structure according to census data and clinic presentations. Fig C Number of outpatient clinic visits for the main diagnoses according to age group and sex. Fig D Proportions referred to hospital among outpatients with specific diagnoses. Fig E Referrals to hospital according to age group. Fig F Proportions prescribed antibiotics for the main diagnoses by sex. Fig G Factors associated with Watch antibiotic prescribing. (DOCX) [file pgph.0004442.s001.docx]

**Title:** High burden of infectious diseases and antibiotic prescribing among patients presenting to primary care in Harare, Zimbabwe

**Authors:** Ioana D. Olaru, Rudo MS Chingono, Fadzaishe Mhino, Celia Gregson, Christian Bottomley, Tsitsi Bandason, Chipo E. Mpandaguta, Karlos Madziva, Rashida A. Ferrand, Michael Vere, Prosper Chonzi, Shungu Munyati, Justin Dixon, Thomas C Darton, Katharina Kranzer

**Table of contents**

**[Fig A](#_Toc190184868)** [Map of Harare representing the clinics included in the study. 2](#_Toc190184868)

**[Table A](#_Toc190184869)** [Number of presentations according to year and clinic 3](#_Toc190184869)

**[Fig B](#_Toc190184870)** [Comparison of population age structure according to census data and clinic presentations 3](#_Toc190184870)

**[Fig C](#_Toc190184871)** [Number of outpatient clinic visits for the main diagnoses according to age group and sex 4](#_Toc190184871)

**[Fig D](#_Toc190184872)** [Proportions referred to hospital among outpatients with specific diagnoses. 4](#_Toc190184872)

**[Fig E](#_Toc190184873)** [Referrals to hospital according to age group 5](#_Toc190184873)

**[Fig F](#_Toc190184874)** [Proportions prescribed antibiotics for the main diagnoses by sex. 7](#_Toc190184874)

**[Fig G](#_Toc190184875)** [Factors associated with Watch antibiotic prescribing 7](#_Toc190184875)

**[Table C](#_Toc190184876)** [Characteristics of patients according to recorded temperature 8](#_Toc190184876)

**[Table D](#_Toc190184877)** [Antibiotic prescribing according to month of presentation 8](#_Toc190184877)

# **Fig A** Map of Harare representing the clinics included in the study.


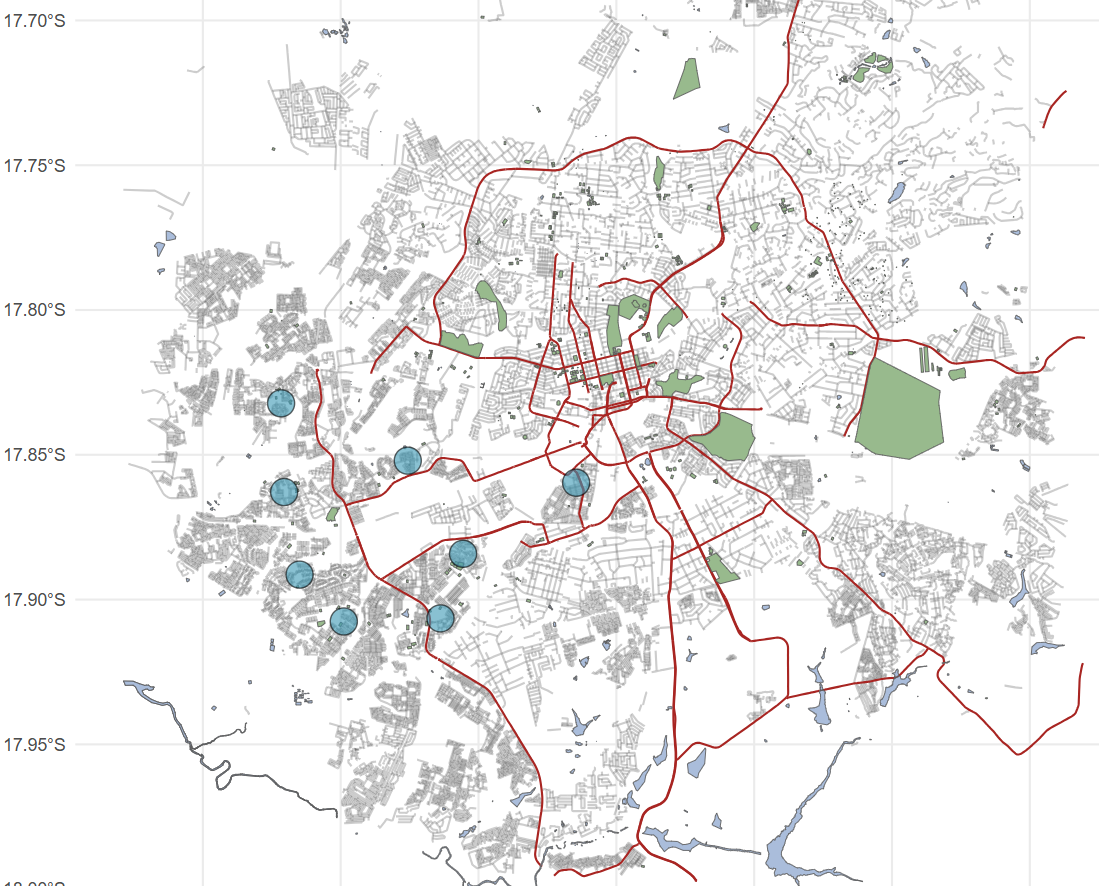


# **Table A** Number of presentations according to year and clinic

| **Clinic** | **Year** | | | | | |
| --- | --- | --- | --- | --- | --- | --- |
|  | **2016** | **2017** | **2018** | **2019** | **2020*** | **2021*** |
| Clinic 1 | 6081 | 2235 | 1961 | 5516 | 856 | 3299 |
| Clinic 2 | 365 | 2896 | 9726 | 5353 | 1926 | 1136 |
| Clinic 3 | 3477 | 3407 | 2784 | 1808 | 1118 | 129 |
| Clinic 4 | 17 | 726 | 13237 | 9840 | 1750 | 787 |
| Clinic 5 | 2589 | 3376 | 4228 | 1649 | 796 | 0 |
| Clinic 6 | 13 | 529 | 5548 | 6919 | 2716 | 1465 |
| Clinic 7 | 6557 | 9122 | 11338 | 6542 | 3159 | 6208 |
| Clinic 8 | 12008 | 9841 | 10166 | 8754 | 2777 | 3150 |

**Considerable service disruptions due to lockdowns and clinic closures occurred during the COVID-19 pandemic*

# **Fig B** Population age structure according to the census data (A) and density plot overlaying patient age and age structure of the population according to census data (B)^1^

| 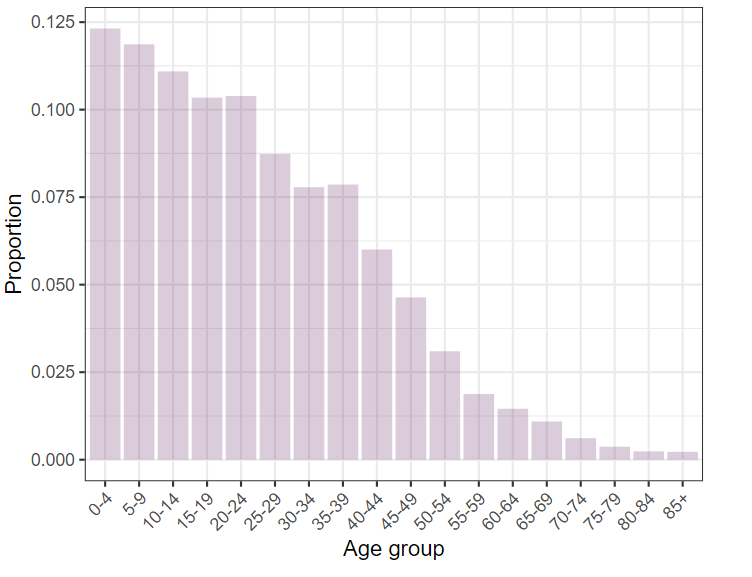 | 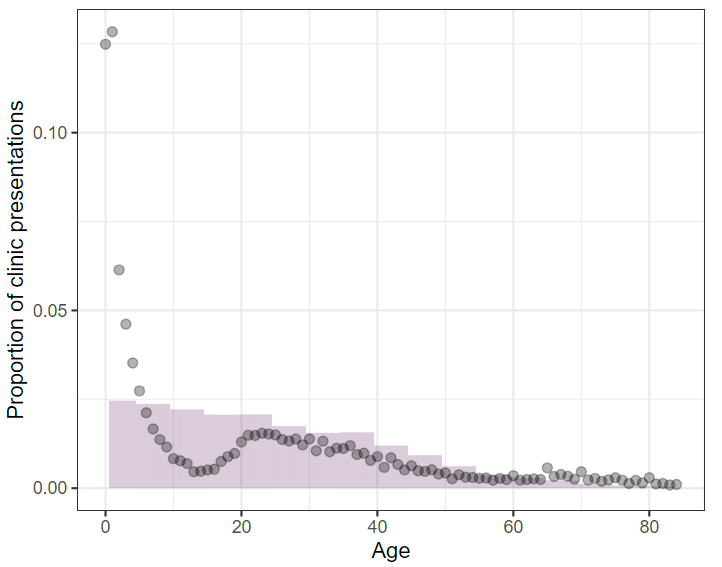 |
| --- | --- |

*Purple: population structure according to census data; grey dots: distribution of clinic attendances*

1. Zimbabwe National Statistics Agency. Zimbabwe 2022 Population and Housing Census. Available from https://www.zimstat.co.zw/census/. Accessed 11 February 2024.

# **Fig C** Number of outpatient clinic visits for the main diagnoses according to age group and sex


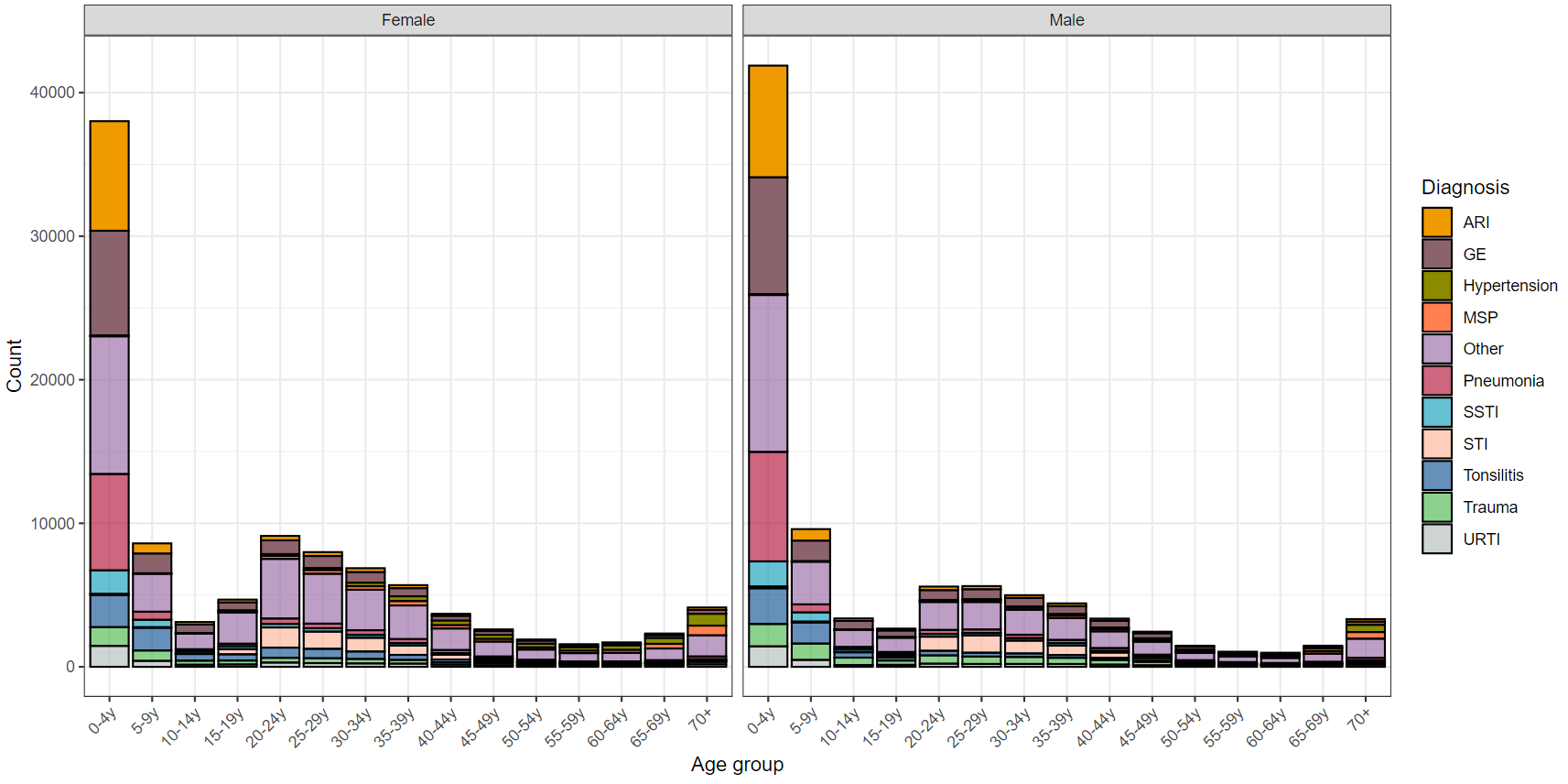


*ARI: acute respiratory infections; GE: gastrointestinal infections; MSP: musculoskeletal pain; SSTI: skin and soft-tissue infections; STI: sexually transmitted infections; URTI: upper respiratory tract infections*

# **Fig D** Proportions referred to hospital among outpatients with specific diagnoses. The x-axis shows the absolute numbers presenting with the specific condition while the y-axis shows the proportion referred among those presenting (male: squares; females: circles).


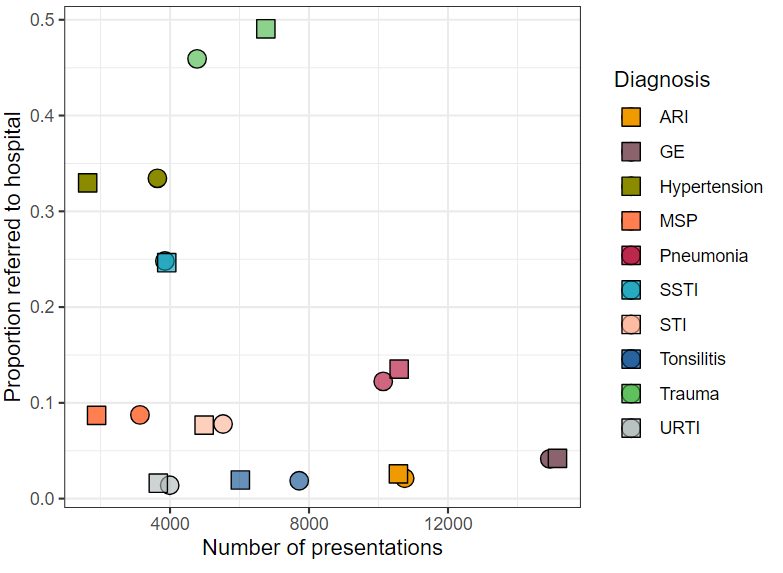


*ARI: acute respiratory infections; GE: gastrointestinal infections; MSP: musculoskeletal pain; SSTI: skin and soft-tissue infections; STI: sexually transmitted infections; URTI: upper respiratory tract infections.*

# **Fig E** Referrals to hospital according to age group for all presentations (A); gastrointestinal infections (B) and pneumonia (C)

| 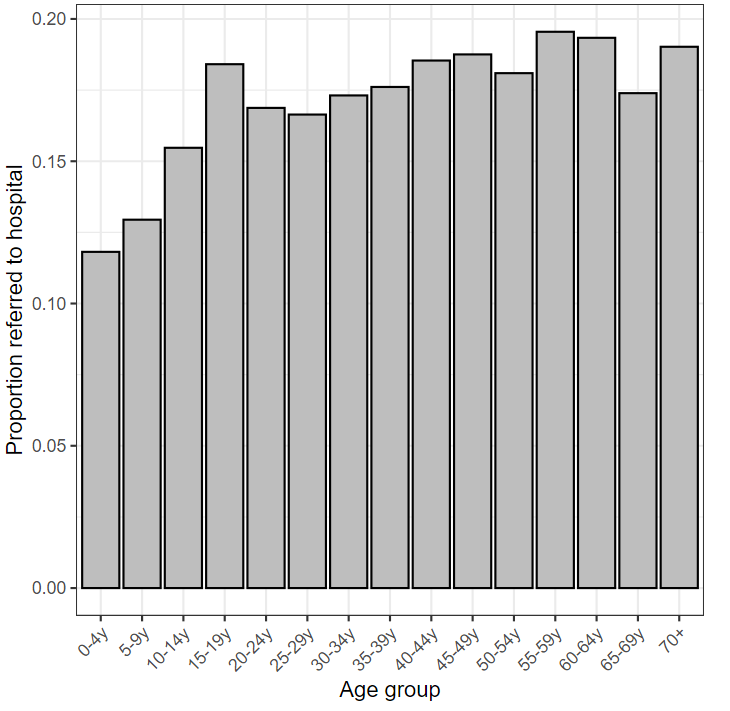 | 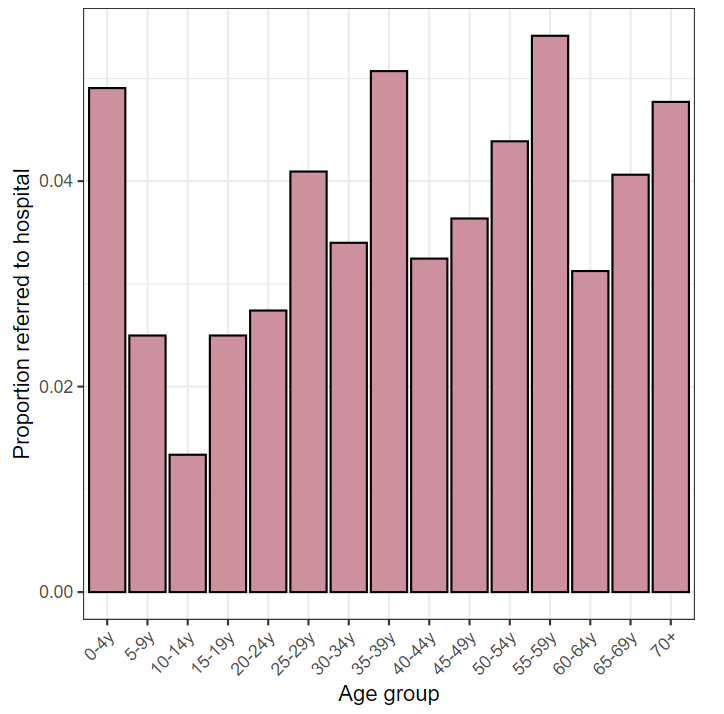 |
| --- | --- |
| 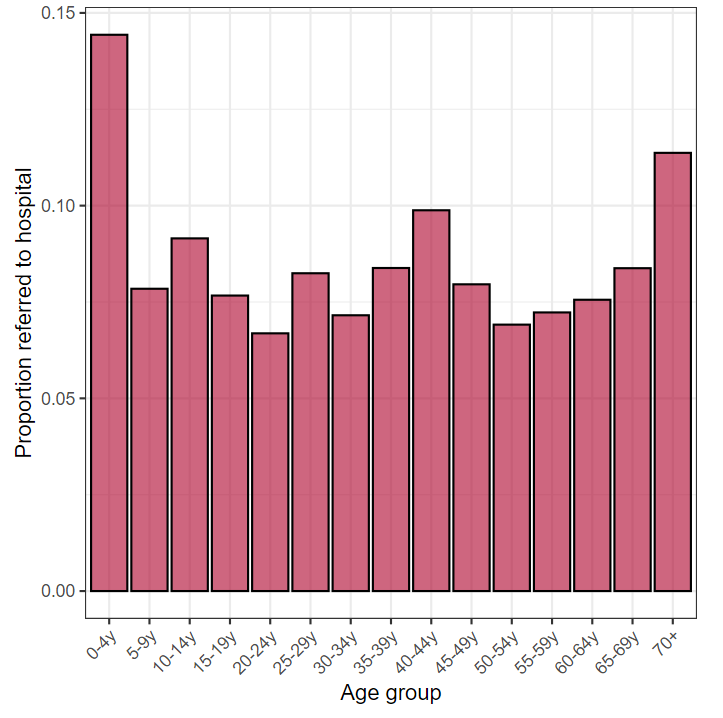 |  |

**Table B** Other diagnoses (includes all patients irrespective of hospital referral who did not receive any other diagnosis)

| **Main diagnostic category** | **Conditions included*** | **Number with the diagnosis** |
| --- | --- | --- |
| Allergic conditions | Allergic rash, allergic conjunctivitis, allergic reactions, urticaria, eczema, drug allergy | 2585 |
| Arthritis | - | 1126 |
| Asthma | - | 333 |
| Cardiovascular, non-infectious | Chronic chest pain, angina pectoris, palpitations | 825 |
| Congestive cardiac failure | - | 132 |
| Dental | Toothache, dental abscess, caries, broken tooth | 574 |
| Diabetes mellitus | - | 266 |
| Ear, nose and throat infections | Chronic mastoiditis, sinusitis, oral ulcers, thrush, gingivitis, stomatitis | 1097 |
| Fever | - | 2154 |
| Gastrointestinal, infectious | Appendicitis, jaundice, acute abdomen | 176 |
| Gastrointestinal, non-infectious | Peptic ulcer disease, epigastric pain, gastric pain, abdominal pain, hyperacidity, dyspepsia, melena, haemorrhoids, gastrointestinal bleeding, constipation, distended abdomen | 3739 |
| Genital, non-infectious | Vaginal bleeding, menorrhagia, ovarian cysts, breast cancer, breast tumour, amenorrhea, breast pain, breast swelling | 563 |
| Headache | - | 4162 |
| Malaria | - | 952 |
| Malnutrition | - | 176 |
| Ocular infections | Conjunctivitis, septic eye, eye infection, trachoma, discharge, eyelid abscess | 1546 |
| Pregnancy associated conditions | Abortion, incomplete abortion, threatened abortion, abdominal pain in pregnancy, discomfort in pregnancy, early pregnancy, hyperemesis, vaginal bleeding | 2235 |
| Rash | Rashes, sores, ringworm, tinea | 2987 |
| Schistosomiasis | - | 145 |
| Seizures | Seizures, epilepsy | 252 |
| Tuberculosis | - | 1088 |
| Typhoid fever | - | 1454 |
| Urinary tract infections | - | 3230 |
| Varicella | - | 1474 |

**the list of conditions included is not exhaustive*

# **Fig F** Proportions prescribed antibiotics among all outpatients (A) with specific diagnoses (excluding those referred to hospital) and among patients aged 10 years and above (B).The x-axis shows the absolute numbers presenting with the specific condition while the y-axis shows the proportion with antibiotic prescriptions among those presenting (male: squares; females: circles).

| 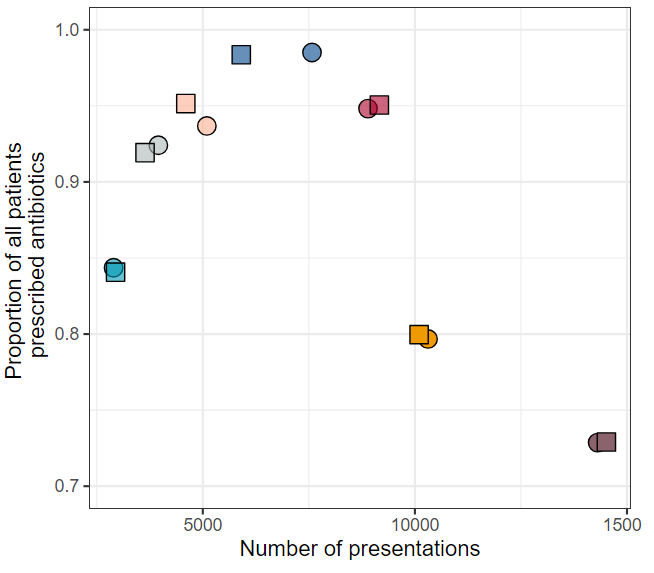 | 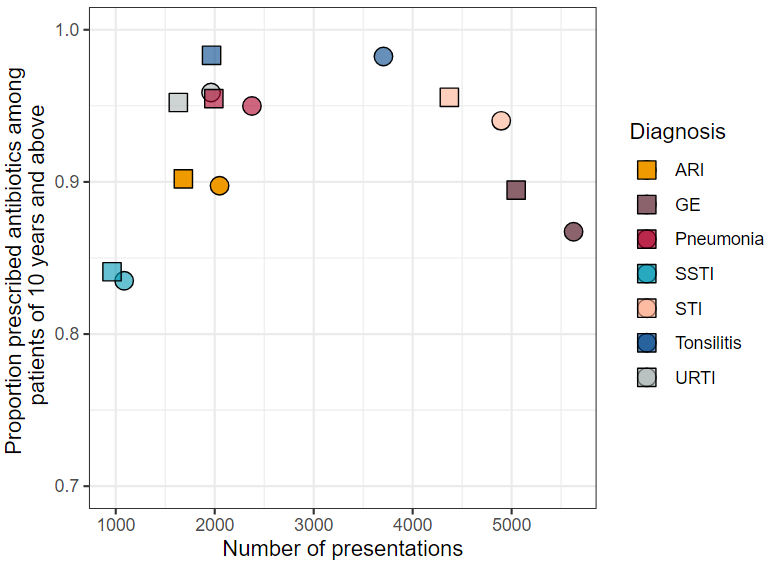 |
| --- | --- |

*ARI: acute respiratory infections; GE: gastrointestinal infections; SSTI: skin and soft-tissue infections; STI: sexually transmitted infections; URTI: upper respiratory tract infections*

# **Fig G** Factors associated with Watch antibiotic prescribing


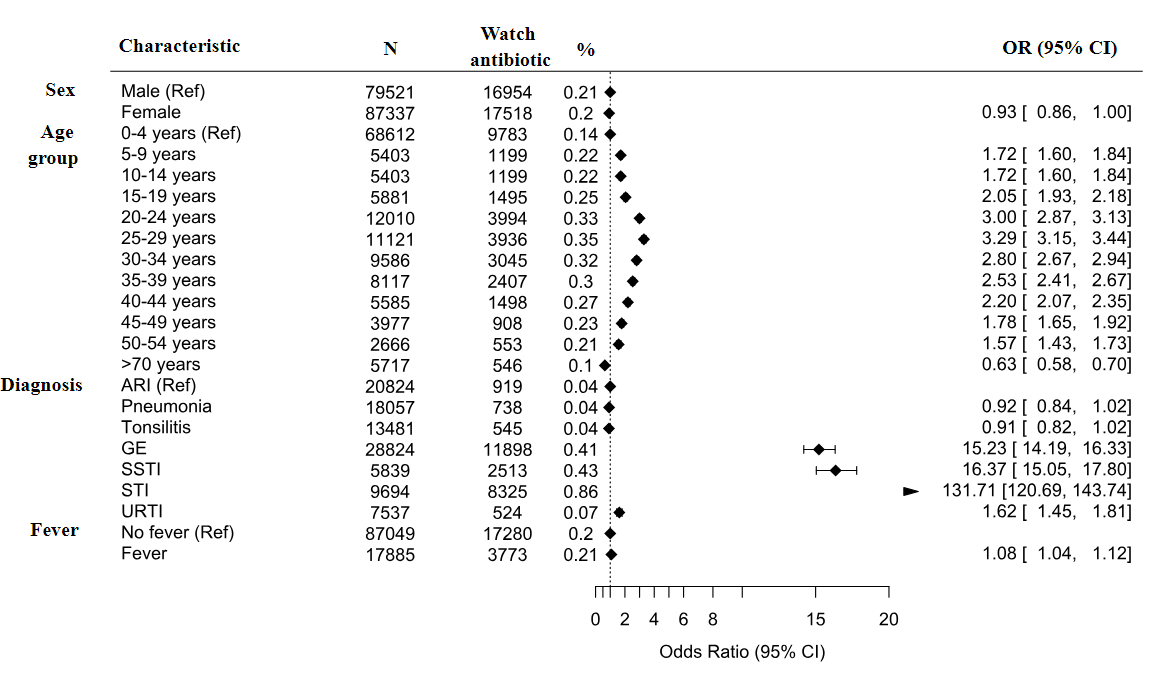
*ARI: acute respiratory infections; GE: gastrointestinal infections; MSP: musculoskeletal pain; OR: odds ratio; SSTI: skin and soft-tissue infections; STI: sexually transmitted infections; URTI: upper respiratory tract infections*

# **Table C** Characteristics of patients presenting to primary care according to recorded temperature among those who were not referred to hospital

| **Characteristic** | **Total with recorded temperature**  **N=104 934** | **Febrile (≥37.5°C)**  **N=17 885** | | **Non-febrile**  **N=87 049** | |
| --- | --- | --- | --- | --- | --- |
|  |  | **Antibiotics**  **N=16 149** | **No antibiotics**  **N=1736** | **Antibiotics**  **N=58 390** | **No antibiotics**  **N=28 659** |
| Age, median (IQR) years§ | 6 (1-28) | 3 (1-10) | 3 (1-26) | 6 (1-28) | 8 (1-33) |
| **Age group (years), n (%)**  0-4  5-9  10-14  15-19  20-24  25-29  30-34  35-39  40-44  45-49  50-54  55-59  60-64  65-69  ≥70 | 49 298 (48.3)  9251 (9.1)  3081 (3.0)  3350 (3.3)  7065 (6.9)  6568 (6.4)  5599 (5.5)  4671 (4.6)  3199 (3.1)  2183 (2.1)  1341 (1.3)  987 (1.0)  999 (1.0)  1512 (1.5)  2903 (3.1) | 9770 (62.1)  1949 (12.4)  474 (3.0)  425 (2.7)  775 (4.9)  634 (4.0)  573 (3.6)  431 (2.7)  255 (1.6)  151 (1.0)  84 (0.5)  56 (0.4)  40 (0.3)  49 (0.3)  70 (0.4) | 887 (52.6)  135 (8.0)  59 (3.5)  64 (3.8)  98 (5.8)  110 (6.5)  91 (5.4)  67 (4.0)  55 (3.3)  38 (2.3)  18 (1.1)  11 (0.7)  8 (0.5)  17 (1.0)  27 (1.6) | 26 153 (46.1)  5318 (9.4)  1822 (3.2)  1881 (3.3)  4288 (7.6)  4129 (7.3)  3407 (6.0)  2817 (5.0)  1900 (3.3)  1255 (2.2)  767 (1.4)  519 (0.9)  509 (0.9)  708 (1.2)  1291 (2.3) | 12488 (44.9)  1849 (6.6)  726 (2.6)  980 (3.5)  1904 (6.8)  1695 (6.1)  1528 (5.5)  1356 (4.9)  989 (3.6)  739 (2.7)  472 (1.7)  401 (1.4)  442 (1.6)  738 (2.7)  1515 (5.4) |
| **Female sex** | 54 362 (51.8) | 7909 (49.0) | 852 (49.1) | 29 941 (51.3) | 15 660 (54.6) |
| **Diagnostic**  Gastroenteritis  Sexually transmitted infection  Acute respiratory infection  Pneumonia  Tonsilitis  Skin and soft-tissue infection  Upper respiratory tract infection  Trauma  Hypertension  Musculoskeletal pain  Other diagnoses | 18 886 (18.0)  5405 (5.2)  13 225 (12.6)  12 539 (11.9)  8808 (8.4)  3850 (3.7)  4576 (4.4)  3439 (3.3)  1255 (1.2)  2338 (2.2)  31 131 (29.7) | 2223 (13.8)  167 (1.0)  2291 (14.2)  3774 (23.4)  2927 (18.1)  225 (1.4)  986 (6.1)  57 (0.4)  7 (0.0)  30 (0.2)  3575 (22.1) | 383 (22.1)  9 (0.5)  149 (8.6)  90 (5.2)  38 (2.2)  22 (1.3)  29 (1.7)  38 (2.2)  25 (1.4)  51 (2.9)  905 (52.1) | 11 004 (18.8)  4921 (8.4)  7955 (13.6)  8077 (13.8)  5762 (9.9)  3013 (5.2)  3196 (5.5)  1528 (2.6)  137 (0.2)  251 (0.4)  12 880 (22.1) | 5276 (18.4)  308 (1.1)  2830 (9.9)  598 (2.1)  81 (0.3)  590 (2.1)  365 (1.3)  1816 (6.3)  1086 (3.8)  2006 (7.0)  13 771 (48.1) |

*§2927 patients missing age; those with unknown referral status were excluded*

# **Table D** Antibiotic prescribing according to month of presentation among patients who were not referred to hospital and excluding those with unknown referral status

| **Month** | **Number of presentations** | **Number prescribed any antibiotics** | **Proportion prescribed any antibiotics** | **Proportion prescribed Access antibiotics** | **Proportion prescribed Watch antibiotics** |
| --- | --- | --- | --- | --- | --- |
| January | 15063 | 10220 | 67.8% | 45.6% | 22.0% |
| February | 16140 | 11627 | 72.0% | 53.7% | 18.2% |
| March | 19189 | 14512 | 75.6% | 58.3% | 17.2% |
| April | 13407 | 9364 | 69.8% | 50.0% | 19.7% |
| May | 13621 | 9430 | 69.2% | 49.0% | 20.2% |
| June | 13931 | 9984 | 71.7% | 52.0% | 19.6% |
| July | 13078 | 9428 | 72.1% | 54.2% | 17.7% |
| August | 12260 | 8472 | 69.1% | 48.6% | 20.4% |
| September | 13257 | 8700 | 65.6% | 43.3% | 22.3% |
| October | 14109 | 9619 | 68.2% | 46.4% | 21.5% |
| November | 12340 | 8729 | 70.7% | 45.2% | 25.5% |
| December | 10463 | 7589 | 72.5% | 45.4% | 27.0% |
