# Supplementary material for: Infectious diseases burden and antibiotic prescribing patterns among primary care patients in Harare, Zimbabwe – a cross-sectional analysis
Source: PLOS Glob Public Health. 2025 Apr 8;5(4):e0004442. doi: 10.1371/journal.pgph.0004442 (PMC11977971; doi:10.1371/journal.pgph.0004442)
Supplement: Attachment — Submitted filename: PLOS Global Public Health_Comment Review.pdf [file pgph.0004442.s002.pdf]

Title: High burden of infectious diseases and antibiotic prescribing among patients presenting to primary care in Harare, Zimbabwe

Author: Ioana D. Olaru

Journals: PLOS Global Public Health

Confidential comments to Editor:

Comments to the Author

1. **Title:** The title does a good job outlining the scope of the study but could be more specific about the design. The phrase "High burden" feels a bit vague, and "antibiotic prescribing" might seem too broad unless the study itself takes a generalized approach. Consider simplifying phrases like "Patients presenting to primary care" to "Primary care patients" for clarity.
2. **Abstract**
   2.1 Lines 52–54: The phrase "high burden of infectious diseases and disparities in access to and use of antimicrobials" feels repetitive Maybe pair it with "All around the world, the majority of antibiotics are prescribed in the outpatient department" or make the distinction.
   2.2 Line 54: The phrase "data from LMIC are scarce" is too general Maybe be sure to indicate the specific gap your study bridges, such as outpatient trends or antibiotic classification.
   2.3 Line 55: The study goal might be more interesting if it states explicitly what is the hole you are filling.
   2.4 Lines 59–62: There is no mention of who chose which records or how statistical analysis was done.
   2.5 Line 66: Be consistent with the numbers and percentages.
   2.6 Line 69: with 68.9% prescription rate it's kind of ok, but how that compares to other countries? Is it exceptionally high or low?
   2.7 Lines 70–75: In the same way, you might add some context to the patterns of AWaRe categories.
   2.8 Lines 79–82: Recommendations like "increasing access to diagnostics" are quite broad. . They would have greater impact if they were accompanied by examples that pertain to the context of your research, and which are attributed to public health implications or interventions.
3. **Introduction**
   Your intro explains well the global problems with infectious disease and AMR. Some notes to fine-tune it:

   3.1 Line 88-90. Some sentences are a bit long, hard to follow. Then, you could break them up into smaller, more comprehensible sentences.

3.2 Line 88, 97. We see the notion of inequality of access and stewardship come up again and again. These would flow better if they were simplified.
3.3 Line 103-109. Global concerns are covered, but not enough is said about the local scenario in Harare. To an extent, it would have been more contextualised if the study had been done with Harare's healthcare, financial or disease constraints in mind.
3.4 Line 103-105. While the lack of studies in the WHO African region is noted, explain why this is a problem (e.g., limited evidence to inform local policies or interventions)

4. **Methods**
    4.1 Line 112-124. While it's great that you included a detailed overview of the clinics, some information (e.g., maternal care services) isn't directly relevant to the focus on acute consultations and antibiotics. Focus more on acute care settings.
    4.2 Line 115- 120. Little information regarding the setting of acute consultation. If you are focusing that information, describe more on the setting of these types of consultation and talk less of other sectors which are not of concern except it has an impact in the study.
    4.3 Line 123. If there are no consultation fees for children, tell us why it applies to your research.
    4.4 Line 126. Write section titles such as "Study population" or "Data collection" instead of "Patients and data".
    4.5 Line 127-130. Time-delay between dates of consultation and data collection requires explanation. Is there a retrospective or a prospective dimension to this?
    4.6 Line 133-135. When you redistributed free-text diagnoses, what did you do with inconclusive or inconsistent data?
    4.7 Line 144. Were there any issues with paper registers, such as partial or legible paper files? Mentioning this would enhance transparency.
    4.8 Line 149. Tell more about the inclusion and exclusion criteria like what you did with the repeat visits.
    4.9 Line 152-155. Were any adjustments made for confounders in the odds ratio calculations? Were comparisons (e.g., age structure) statistically tested? If so please describe it clearly.
    4.10 Line 153. Could give more justification why specific metrics (e.g., medians, IQRs) were chosen instead of means or other measures.
    4.11 Line 158-161: Although individual consent was waived, briefly justify why this was ethical and appropriate for the study design (e.g., anonymization, minimal risk to participants).
5. **Results**
    You have detailed results that can be cleaned up for more impact and clarity:
    5.1 General comments: Figures and tables are referenced without descriptive summaries. Readers might not immediately understand what these visuals contribute.
    5.2 Line 168-174. All the statistics are too many to digest. Highlight the most significant findings and point readers to tables or figures for the rest.

5.3 Line 171-176. Avoid repetition. The words "acute respiratory infections such as pneumonia and gastrointestinal infections were extremely prevalent in young children" (for example) are repeated.
5.4 Line 177-182. Outline why 10–20 year olds don't show up in clinic visits. Might it be culture, lesser burden of disease or alternative therapies?
5.5 Line 183-185. Outline why sex differences mentioned later is not integrated in the main result for patient characteristics.
5.6 Line 197-207. Referral rate without asking why certain diseases (e.g., pneumonia) received more referrals. Match these with clinical severity or diagnostic Gaps.
5.7 Line 203-205. The text states that referral rates for gastrointestinal infections and pneumonia were "similar across age-groups" but does not elaborate on why younger children were exceptions as indicated later. Quick question. Any information on what happen to the referral cases. Any documentation? It will be good to know the follow up. if not, you can insert it as a limitation because it poses an issue related to the significance of the referral rates.
5.8 Line 213-219. Considering that 60.5% of all patients were prescribed antibiotics and those not referred 68.9% prescribed. Its kinds of give the same information. It will be good to make clearer and referred to tables and figures for further breakdowns. Select what information you want to write specifically.
5.9 Line 235-246; While the AWaRe framework is used, the implications of the findings (e.g., high use of 'Access' and 'Watch' antibiotics) are not discussed. Why is the low prescription of 'Reserve' antibiotics significant? Does this reflect appropriate stewardship or limited availability?
5.10 Line236-237: Ciprofloxacin accounts for 65.1% of 'Watch' prescriptions, yet the reason for its regular use is not discussed. Provide a little more explanation on the main reason for this prescription
5.11 Line 240-246. Respiratory infections are grouped inconsistently (e.g., "acute respiratory infections," "upper respiratory tract infections"). This may bring confusion. Standardise across all findings
5.12 Line 242-246. Assess more critically whether high respiratory infection and gastroenteritis prescribing rates are in line with the clinical practice or indicate overprescription.
5.13 Line 254-257. The section notes that females were less likely to receive antibiotics but does not explore potential reasons. Any reason?
5.14 Line 255. Fever is mentioned as influencing prescriptions but is not quantified or explained. How significant was this factor compared to age or sex?

6. Discussion

The discussion effectively synthesizes the findings, contextualizes them in relation to existing literature, and highlights broader implications for policy and public health. However, there are specific areas where clarity, structure, and focus can be improved.
General comments:

- Key points about AMR, diagnostics, and antibiotic prescribing are spread across multiple paragraphs, reducing their impact. Consolidate related themes (e.g.,

challenges with diagnostics, implications of high antibiotic prescribing rates) into focused subsections.

- The discussion includes lengthy tangential information, such as demographic transitions and urbanization, which detract from the main findings. Briefly acknowledge these broader trends but focus on study-relevant insights.
- Although the results highlight gender differences in antibiotic prescriptions, this is not addressed in the discussion.
- Explicitly compare the results to prior studies to highlight similarities, differences, and contributions. Several sentences are not related to the study.

6.1 Line 266-268. The study's scale and duration are highlighted several times. Rather emphasize how this large dataset provides unique insights into prescribing trends and disease burden.

6.2 Line 273- 279. Explain diagnostic gaps causing overprescription and propose solutions such as point-of-care tests.

6.3 Line 277. The statement "only 19% of people in LMIC have access to diagnostics" is too broad and lacks specificity to Zimbabwe.

6.4 Line 282-285. There are high prescription rates for respiratory and diarrhoea which are not critically evaluated. Any systemic factors (e.g., diagnostic gaps, clinical guidelines) which drive these trends? How do they align with the guidelines.

6.5 Line 290-295. If not published why discussed it. Please describes clearly, it’s relevance to the study.

6.6 Line 305-314. Although the discussion mentions that most diarrheal episodes do not require antibiotics, it fails to evaluate how prescribing could be reduced. It will be interested to suggest interventions like treatment guidelines, Trainings programs etc.

6.7 Line 315-331. The approach for managing STIs is described but not analyzed for its impact on overprescription or resistance. Discuss the risk in management, including overtreatment and missed diagnoses, and suggest alternatives like targeted diagnostic tools.

6.8 Line 329. The severe typhoid outbreaks during the study period are mentioned but not connected to prescribing practices or broader public health responses. Explain more on its impact.

6.9 Line 355- 361. Could you discuss how the double burden of NCDs and infectious diseases complicates antibiotic stewardship efforts.

6.10 Line 366-367. The retrospective design is noted as a limitation, but potential biases in how healthcare workers diagnose and prescribe are not explored.

6.11 Line 368, if you mentioned the private sector, please explain clearly the underestimation on overall antibiotic use.

6.12 Line 370 372. The lack of data on dosage and duration is mentioned but not analyzed for its impact on the findings. Highlight how this affects the assessment of prescribing appropriateness.

7. Conclusion

7.1 Line 390. Include a heading clearly for conclusion

7.2 Lines 391–395: Focus on what your study is different, like the large dataset and its application to LMIC contexts.

7.3 Lines 391–395: Connect your results to other global issues such as AMR and provide solutions, for example stewardship programmes for Harare.

8. **Limitation of the study**

   8.1 Please clearly describe this in the study because it has been noted several times in different sections of the research.
