# Supplementary material for: Infectious diseases burden and antibiotic prescribing patterns among primary care patients in Harare, Zimbabwe – a cross-sectional analysis
Source: PLOS Glob Public Health. 2025 Apr 8;5(4):e0004442. doi: 10.1371/journal.pgph.0004442 (PMC11977971; doi:10.1371/journal.pgph.0004442)
Supplement: Attachment — Submitted filename: response to reviewers.docx [file pgph.0004442.s003.docx]

12-January-2025

**Response to reviewer and editorial comments**

Dear Dr Saravanos,

Thank you for reviewing our manuscript, for providing comments, and for giving us the opportunity to resubmit an improved version. We have addressed all the comments and provide a point-by-point response in the table below.

We hope that you will consider this version suitable for publication. If we can provide further information, please do not hesitate to contact us.

Yours sincerely on behalf of the authors,

Ioana D Olaru

| **Comments** | **Response to comments** |
| --- | --- |
| **Editorial comments** |  |
| Please provide separate figure files in .tif or .eps format. | We have included the figures in .eps format |
| We noticed that you used "unpublished data" in the manuscript. We do not allow these references, as the PLOS data access policy requires that all data be either published with the manuscript or made available in a publicly accessible database. Please amend the supplementary material to include the referenced data or remove the references. | At the time of writing, we anticipated that the data would be published. We have now removed the statement. |
| **REVIEWER 1** |  |
| Title: The title does a good job outlining the scope of the study but could be more specific about the design. The phrase “High burden” feels a bit vague, and "antibiotic prescribing" might seem too broad unless the study itself takes a generalized approach. Consider simplifying phrases like “Patients presenting to primary care” to “Primary care patients” for clarity. | We have changed the title of the manuscript as suggested. It now reads:  *“Infectious diseases burden and antibiotic prescribing patterns among primary care patients in Harare, Zimbabwe – a cross-sectional analysis”* |
| Abstract  Lines 52–54: The phrase "high burden of infectious diseases and disparities in access to and use of antimicrobials" feels repetitive Maybe pair it with "All around the world, the majority of antibiotics are prescribed in the outpatient department" or make the distinction. | We have revised the sentences in the introduction to avoid repetitions and combined the two sentences. It currently reads:  *“Low- and middle-income countries (LMIC) continue to experience a high burden of infectious diseases and disparities in access to and use of antimicrobials, yet data on antibiotic prescribing in outpatient settings, where the majority of global prescriptions occur, remain scarce.”* (lines 52-54) |
| Line 54: The phrase "data from LMIC are scarce" is too general Maybe be sure to indicate the specific gap your study bridges, such as outpatient trends or antibiotic classification. | This section had to be summarised for the purposes of the abstract. The scarcity of the data is addressed in more detail in the manuscript text and references to published literature are provided (highlighting the lack of data – lines 104-110). |
| Line 55: The study goal might be more interesting if it states explicitly what is the hole you are filling. | The objective has been revised to reflect the gap that is being filled by the study (following on from the previous sentence)  *“The objective of this study is to provide data on diagnoses and antibiotic prescriptions among primary care patients in Harare, Zimbabwe.”* (lines 55-56) |
| Lines 59–62: There is no mention of who chose which records or how statistical analysis was done. | A clarification on how clinic selection was conducted was added  *“Clinics were selected based on the population they served and the availability of records.”* (lines 60-61)”  All records identified from outpatients in participating clinics were included. Given that the statistical analysis is largely descriptive, we have not included the information in the abstract for the purpose of brevity. |
| Line 66: Be consistent with the numbers and percentages. | We have added the counts in the parentheses. The sentence now reads: *“The median patient age was 9 years and 52.5% (105,035/199,880) were female.”* (lines 66-67) |
| Line 69: with 68.9% prescription rate it’s kind of ok, but how that compares to other countries? Is it exceptionally high or low? | A sentence was added to the introduction on antibiotic prescribing in other settings. To improve consistency in reporting, the figure was revised.  *“Antimicrobial use in the WHO African region remains relatively understudied. Only 15 studies were identified by a systematic review of studies on antimicrobial prescriptions in primary care published between 2010 and 2020, most with relatively small sample sizes.[5] However, antibiotic prescriptions were generally high and wide-ranging from 20 to 88%.[5]”* (lines 104-107). These figures are higher than those reported from high-income settings. |
| Lines 70–75: In the same way, you might add some context to the patterns of AWaRe categories. | Further contextualisation could not be added here because of limits on word count for the abstract. The AWaRe classification and use according to diagnosis are discussed throughout the manuscript text.  *“Antibiotic prescriptions were categorised into groups according to the AWaRe (Access, Watch and Reserve) classification.[12] Antibiotics belong to the ‘Access’ group if they are narrow spectrum and have a good safety profile. ‘Watch’ antibiotics have a broader spectrum and a higher resistance potential, while ‘Reserve’ antibiotics are last-choice used to treat multidrug resistant infections.[13]* (lines 152-156)  *“Most outpatient presentations resulted in antibiotic prescriptions, of which 70% belonged to the ‘Access’ group which is lower than the suggested 90% ‘Access’ antibiotics for primary care.[14] Most ‘Watch’ group antibiotics were prescribed for diarrheal diseases and sexually transmitted infections. As expected, ‘Reserve’ antibiotics were not prescribed given that antibiotics belonging to this group are costly, often require intravenous administration, and are not usually available in the primary care setting.”* (lines 291-296) |
| Lines 79–82: Recommendations like "increasing access to diagnostics" are quite broad. . They would have greater impact if they were accompanied by examples that pertain to the context of your research, and which are attributed to public health implications or interventions. | We agree that the phrasing on diagnostics is broad but limits on word count for the abstract do not allow to discuss these in more detail. The generalisation means to cover multiple aspects of diagnostics that would be needed such as tests for differentiation between viral and bacterial infection (such as CRP or other biomarkers); tests for specific infections (such as typhoid fever, influenza, etc.). These issues are discussed in detail in the manuscript lines 355-371. |
| Introduction  Line 88-90. Some sentences are a bit long, hard to follow. Then, you could break them up into smaller, more comprehensible sentences. | The sentences have been shortened as recommended. They now read:  *“Globally, antimicrobial use has increased by almost 50% over the last two decades. However, considerable disparities in access and use persist.”* (lines 87-89) |
| Line 88, 97. We see the notion of inequality of access and stewardship come up again and again. These would flow better if they were simplified. | We meant to use the two concepts differently. On one hand to point out differences in access to antibiotics between settings and on the other hand to state the need for contextualised stewardship interventions for optimising antibiotic use. |
| Line 103-109. Global concerns are covered, but not enough is said about the local scenario in Harare. To an extent, it would have been more contextualised if the study had been done with Harare’s healthcare, financial or disease constraints in mind. | There are no studies on antibiotic use in primary care from Zimbabwe. Additional information on the Zimbabwean context were added:  “*Infectious diseases are common in Zimbabwe but data on clinic attendances and antibiotic use are lacking. Zimbabwe also has a high prevalence of HIV of 12.9% among adults [9] while 21.6% and 2.9% children under the age of 5 have either stunting or wasting,[10] all of which increase the risk and severity of infections.”* (lines 109-112)  This analysis makes use of routine clinical data and focuses on the clinical presentations and antibiotic use. Further published work by our group discusses constraints of the healthcare setting (including financial) and focuses on other infections and syndromes. |
| Line 103-105. While the lack of studies in the WHO African region is noted, explain why this is a problem (e.g., limited evidence to inform local policies or interventions) | A sentence was added as suggested to explain the implications of limited data.  *“This results in limited evidence for informing local policies and interventions aimed at reducing the burden of infections and optimising antibiotic use.”* (lines 107-108) |
| Methods  Line 112-124. While it’s great that you included a detailed overview of the clinics, some information (e.g., maternal care services) isn’t directly relevant to the focus on acute consultations and antibiotics. Focus more on acute care settings. | We have now removed the additional information on the clinics which is not directly relevant to the analysis. |
| Line 115- 120. Little information regarding the setting of acute consultation. If you are focusing that information, describe more on the setting of these types of consultation and talk less of other sectors which are not of concern except it has an impact in the study. | The acute care services from these clinics are similar to those provided by GPs (family doctors) in high-income settings and by emergency departments (e.g. hospital emergency room). The clinics also act as a first point of care and triage point for patients with acute conditions (decision to issue a prescription or refer to hospital). Clarification on what type of acute care is provided were added:  *“The clinics act as a first point of contact with a healthcare provider for these communities.”* (lines 122-123)  *“Primary care is provided by trained nurses who conduct consultations and prescribe treatment for a wide range of conditions including communicable and non-communicable diseases.”* (lines 125-127) |
| Line 123. If there are no consultation fees for children, tell us why it applies to your research. | Consultation fees are relevant because they influence access to healthcare. For example, adults who have to pay will be less likely to present for minor conditions while clinic fees would not constitute a barrier for small children. A clarification was added:  *“Consultation fees impact access to health care with individuals required to pay being less likely to present for minor conditions.”* (lines 130-131) |
| Line 126. Write section titles such as "Study population" or "Data collection" instead of "Patients and data". | The section was renamed as “Data collection” according to the suggestion. |
| Line 127-130. Time-delay between dates of consultation and data collection requires explanation. Is there a retrospective or a prospective dimension to this? | The data were always retrospectively collected. The health care workers completed a register book after which it could be used to collect data (to avoid disruptions of clinical activities). The two sets of time intervals were a requirement from the PLOS GPH editorial team which asked to provide both the period when patients presented and the period for data collection.  A clarification that the data were collected retrospectively was added  *“Data were collected retrospectively between 6th January 2020 and 16th April 2022 and included…”* (lines 138-139) |
| Line 133-135. When you redistributed free-text diagnoses, what did you do with inconclusive or inconsistent data? | If the information was insufficient for a diagnosis to be redistributed (e.g. diagnoses such as “undefined illness”, “fever of unknown origin”), then the diagnosis was allocated to the “other” category. The dataset contains minimal clinical information, and healthcare workers may have had different reasons to prescribe medicines that may not be first-choice for the condition at hand. For these reasons we refrained from recategorizing inconsistent information (insufficient information). |
| Line 144. Were there any issues with paper registers, such as partial or legible paper files? Mentioning this would enhance transparency. | Legibility was usually not an issue and where the writing was unclear, we consulted within the team. Research staff were based in the clinics so they could also ask the regular clinic staff in case of illegibility. Whenever the research staff was unsure of the exact spelling, they were asked to record as read. The information was then recoded by a medical doctor under the correct categorisation. Given that the diagnoses and medicines prescribed were short and repetitive, incorrect entries could be reclassified reliably. A sentence was added to address this point:  *“Because the range of diagnoses recorded was relatively limited, typographical errors could be easily identified and corrected.”* (lines 145-146)  Consultations were not always consistently recorded which is listed as one of the limitations of our study.  *“Our study is limited by its retrospective approach to data collection, the limited number of variables for which data were collected and incomplete recording often experienced in routine paper-based clinical registers”* (lines 411-414) |
| Line 149. Tell more about the inclusion and exclusion criteria like what you did with the repeat visits. | There were no inclusion or exclusion criteria for outpatients presenting to the primary care clinic. Patients presenting to other departments (HIV and tuberculosis clinics, antenatal care, maternity and well child clinics) were not included.  *“Data on all recorded patient consultations from the outpatient department conducted between 1st January 2016 and 31st December 2022 were collected from clinic paper registries. Patients presenting to the tuberculosis and HIV clinics as well as those from the antenatal-, maternity and well-child clinics were excluded.”* (lines 135-138)  *“The population included was as follows: all patients presenting to the outpatient department for the analysis of diagnoses…”* (lines 165-166)  The records did not contain information on whether the consultations were initial or repeat visits. A clarification was included in the limitations:  *“Further, we could not determine recurrent attendances for the same individuals or conditions a…”* (lines 425-426) |
| Line 152-155. Were any adjustments made for confounders in the odds ratio calculations? Were comparisons (e.g., age structure) statistically tested? If so please describe it clearly. | The odds ratios presented in the manuscript are not adjusted. Because this is a minimal variable dataset, we chose not to focus on a specific study question and conduct a classical multivariable analysis while investigating and adjusting for potential confounders. The aim of this manuscript was to report on the spectrum of diagnoses and antibiotic prescribing descriptively.  Further, because of the size of the dataset, small differences between groups would be statistically significant. For this reason we chose to present differences in terms of odds ratios with confidence intervals (instead of p-values) as seen in Figure 3 and Supplement Figure S7. For the example of age and compared to the reference age group (0-4 years), all the other age groups would be considered as having a statistically significant difference (confidence intervals not crossing 1). This is described in the methods: *“Unadjusted odds ratios and 95% confidence intervals were computed.”* (lines 170-171) |
| Line 153. Could give more justification why specific metrics (e.g., medians, IQRs) were chosen instead of means or other measures. | Age was the only variable presented as a median with IQR and it does not have a normal distribution.  The information was added in the text  *“§age does not have a normal distribution in the population and is presented as median with IQR”* (lines 229-230) |
| Line 158-161: Although individual consent was waived, briefly justify why this was ethical and appropriate for the study design (e.g., anonymization, minimal risk to participants). | This is a retrospective study of routine medical records and includes patients who presented to the clinic prior to data collection. All data was annonymised at the point of collection. A rationale was provided in the manuscript:  “*… only anonymised data were entered in the electronic database and individuals were not identifiable to researchers accessing the database or conducting the analysis.”* (lines 160-162)  *“This was a retrospective study of routine medical records…”* (line 177) |
| Results  General comments: Figures and tables are referenced without descriptive summaries. Readers might not immediately understand what these visuals contribute. | Descriptions were added to the figures and tables in the methods and results sections. |
| Line 168-174. All the statistics are too many to digest. Highlight the most significant findings and point readers to tables or figures for the rest. | The sentence was simplified as suggested and some of the statistics removed for improved readability. It now reads *“When comparing the age distribution of those presenting to primary care clinics with the general population [11], children under the age of 5 years were overrepresented (comprising 41% of the clinic presentations) while 10–20-year-olds were underrepresented...”* (lines 184-187) |
| Line 171-176. Avoid repetition. The words "acute respiratory infections such as pneumonia and gastrointestinal infections were extremely prevalent in young children" (for example) are repeated. | We have reviewed the results section for repetitions and shortened the sentence on acute infections in children. The section reports on overall causes on presentation and separately on presentations among children under the age of five. We have kept the revised sentence because we felt it is important to report on the burden in this specific population as pneumonia and diarrheal disease are major causes of death in children under the age of five. |
| Line 177-182. Outline why 10–20 year olds don’t show up in clinic visits. Might it be culture, lesser burden of disease or alternative therapies? | This is likely due to a combination of factors such as lower burden of disease in this population as well as less access (as compared to children under the age of 5) because of having to pay clinic fees.  A sentence was also added to the discussion:  *“Older children and adolescents presented to the clinics less frequently likely due to a lower burden of disease in the population and limitations in access because of having to pay clinic fees.”* (lines 403-405) |
| Line 183-185. Outline why sex differences mentioned later is not integrated in the main result for patient characteristics. | We have added the differences in diagnoses by sex to the main manuscript by combining figure 1 with former figure S4. (the figure currently has 4 inlays. A figure on the differences in antibiotic prescribing for the main diagnoses by sex was also added (Supplement Figure S6). |
| Line 197-207. Referral rate without asking why certain diseases (e.g., pneumonia) received more referrals. Match these with clinical severity or diagnostic Gaps. | Unfortunately, we were unable to assess pneumonia severity (or severity for other conditions) as this information was not consistently documented in the routine medical records. This reflects the setting of busy and often understaffed primary clinics from a low resource setting.  The high referrals for pneumonia are likely due to the population served: high attendance in children under the age of five (14% of deaths in this population are because of pneumonia; UNICEF Data for Zimbabwe), the high prevalence of HIV (13% in the general population) and the inability to conduct any diagnostic testing in the primary care setting (e.g. chest X-rays, CRP).  A sentence was added to the discussion commenting on pneumonia referrals:  *“Patients with pneumonia were frequently referred likely due to the high proportion of young children among the presentation, the high burden of HIV in the population and the lack of access to diagnostics.”* (lines 335-339)  And on the limited data available for assessing severity  *“…the limited number of variables for which data were collected and incomplete recording often experienced in routine paper-based clinical registers.”* (lines 411-413) |
| Line 203-205. The text states that referral rates for gastrointestinal infections and pneumonia were "similar across age-groups" but does not elaborate on why younger children were exceptions as indicated later. Quick question. Any information on what happen to the referral cases. Any documentation? It will be good to know the follow up. if not, you can insert it as a limitation because it poses an issue related to the significance of the referral rates. | The text was clarified to highlight the more frequent referrals among young children. It now reads:  *“…apart from children <5 years who were more frequently referred for both pneumonia (14.4%; 2,055/14,238) and gastrointestinal infections (4.9%; 751/15,307; Figure S5).”* (lines 223-225)  *“Children under the age of five with pneumonia and diarrheal disease were more commonly referred due to the increased risks of severe outcomes in this age group.”* (lines 337-339)  No information was available on whether patients followed or not the referral recommendations. We added a sentence to the limitations:  *“Further, we could not determine recurrent attendances for the same individuals or conditions and whether patients followed referral recommendations for severe illnesses.”* (lines 425-427) |
| Line 213-219. Considering that 60.5% of all patients were prescribed antibiotics and those not referred 68.9% prescribed. Its kinds of give the same information. It will be good to make clearer and referred to tables and figures for further breakdowns. Select what information you want to write specifically. | We have removed the redundant information and made reference directly to Table 1.  The sentence now reads:  *„Among those who were not referred to hospital, antibiotics were prescribed in 70.5% (117,674/166,858) of patients.”* (lines 234-235) |
| Line 235-246; While the AWaRe framework is used, the implications of the findings (e.g., high use of ‘Access’ and ‘Watch’ antibiotics) are not discussed. Why is the low prescription of ‘Reserve’ antibiotics significant? Does this reflect appropriate stewardship or limited availability? | We have added comments on the use of Access antibiotics: *“Most outpatient presentations resulted in antibiotic prescriptions, of which 70% belonged to the ‘Access’ group which is lower than the suggested 90% ‘Access’ antibiotics for primary care”* and on Reserve antibiotics: *“As expected, ‘Reserve’ antibiotics were not prescribed given that antibiotics belonging to this group are costly, often require intravenous administration, and are not usually available in the primary care setting.”* (lines 291-296) |
| Line236-237: Ciprofloxacin accounts for 65.1% of ‘Watch’ prescriptions, yet the reason for its regular use is not discussed. Provide a little more explanation on the main reason for this prescription | Ciprofloxacin was mainly prescribed for gastroenteritis. In addition, during the data collection period covered by the survey, Harare was experiencing a typhoid outbreak. Typhoid is very difficult to diagnose based on clinical symptoms and ciprofloxacin was the treatment of choice as recommended by the national guidelines. The use of ciprofloxacin was clarified in the discussion:  *“‘Watch’ antibiotics, mainly ciprofloxacin, were frequently prescribed for gastroenteritis in our study.”* (lines 310-311)  *“In addition, ciprofloxacin is the recommended treatment for typhoid fever and Harare was experiencing a typhoid outbreak during the time covered by the data collection period.”* (lines 314-316) |
| Line 240-246. Respiratory infections are grouped inconsistently (e.g., "acute respiratory infections," "upper respiratory tract infections"). This may bring confusion. Standardise across all findings | We agree that there are overlaps between the diagnostic groups. However, we reported the diagnostics as documented in the medical records by the healthcare workers. As the data contains minimal clinical information and no diagnostic test results, it would have been challenging to reclassify the diagnostic groups.  We have added a clarification on the methods on the diagnosing relating to respiratory infections.  We have added a clarification in the methods to better define the categories:  *“Respiratory tract infections included the following diagnoses: upper respiratory tract infections (URTI), lower respiratory tract infections (LRTI), respiratory tract infections (RTI); acute respiratory infections (ARI), bronchitis, pneumonia, sinusitis, pharyngitis, tonsilitis, otitis, and COVID-19.“* (lines 146-149) |
| Line 242-246. Assess more critically whether high respiratory infection and gastroenteritis prescribing rates are in line with the clinical practice or indicate overprescription. | We fully agree that a lot of the prescriptions in this setting were unnecessary. Comments to emphasize the overprescription of antibiotics were added for respiratory infections *“…, antibiotics were overprescribed with >90% of patients receiving a prescription.”* (lines 300-301)  And for gastrointestinal infections: *“Gastroenteritis was an extremely common cause of presentation across all age groups and 70% of diarrheal episodes were treated with antibiotics, although antibiotics are not indicated for most diarrhoea cases.”* (lines 301-303)  However, as indicated in the limitations section, we refrained from categorising individual prescriptions in terms of appropriateness because of the very limited clinical information. |
| Line 254-257. The section notes that females were less likely to receive antibiotics but does not explore potential reasons. Any reason? | This is an overall observation which does not hold true for the most common infection diagnoses. In order to illustrate this, we have added two new graphs to the supplement which show a direct comparison in antibiotic prescriptions by sex for the most common diagnoses among all age groups and among patients ≥10 years. (Supplement Figure S6)  We also clarified this point in the results: *“Overall, females were 15% (95%CI 10-21) less likely to receive antibiotic prescriptions than men although there were no substantial differences for the most common diagnoses”* (lines 275-277) |
| Line 255. Fever is mentioned as influencing prescriptions but is not quantified or explained. How significant was this factor compared to age or sex? | Patients with fever were four-times as likely to be prescribed antibiotics than those without. In contrast, differences in age and sex played a less important role. To clarify we added a sentence defining fever to the methods:  *“Fever was defined as a temperature of 37.5°C and above as documented in the records.”*  And a sentence to the increased likelihood of antibiotics in the results: *“Patients with fever were 4.6 times (95%CI 4.3-4.8) as likely to receive antibiotics.”* (lines 278-279) |
| Discussion  General comments: - Key points about AMR, diagnostics, and antibiotic prescribing are spread across multiple paragraphs, reducing their impact. Consolidate related themes (e.g., challenges with diagnostics, implications of high antibiotic prescribing rates) into focused subsections. | We have reorganised the discussion section according to broader themes as suggested. The themes include: burden of infection and antibiotic prescribing for the main infectious syndromes; severity of infections and referrals; solutions for reducing prescribing and infection (immunizations, diagnostics, WASH, stewardship); prescribing and aging. |
| The discussion includes lengthy tangential information, such as demographic transitions and urbanization, which detract from the main findings. Briefly acknowledge these broader trends but focus on study-relevant insights. | This section was made more concise, as suggested. We kept the comments on urbanisation and changes in demography because these are anticipated to have an important impact on infectious diseases and on antibiotic prescribing in the future. These link to the need for strengthening preventive measures which are also discussed (e.g. vaccination, access to WASH). |
| Although the results highlight gender differences in antibiotic prescriptions, this is not  addressed in the discussion. | Thank you for the comment. As shown for previous comments, while an overall difference in antibiotic prescriptions was observed between women and men, this difference was not substantial when considering the main infectious diagnoses. To clarify this point we added a sentence to the results: *“Overall, females were 15% (95%CI 10-21) less likely to receive antibiotic prescriptions than men although there were no substantial differences for the most common diagnoses”* and Figure S6 in the supplement also explores differences in prescribing by sex for the main diagnosis categories. |
| Explicitly compare the results to prior studies to highlight similarities, differences, and contributions. Several sentences are not related to the study. | We have revised the introduction and discussion section and added comparisons to other studies published from Africa: *“However, antibiotic prescriptions were generally high and wide-ranging from 20 to 88%.[5] “* (lines 106-107) and from studies conducted in Zimbabwe:  *“A study conducted in Zimbabwe among individuals aged 16 to 24 years who underwent testing for Chlamydia trachomatis and Neisseria gonorrhoeae found that almost 20% of women and 10% of men had a positive test for sexually transmitted infections. However, only 40% of those reporting symptoms of sexually transmitted infections had a positive test, and most of those testing positive were asymptomatic.*[22]” (lines 326-330) This point was made relating to over- and under-treatment for STIs and the need for diagnostics.  *“Further, at the time of this study, several districts in Harare were experiencing a severe typhoid fever outbreak with almost 20% of blood cultures from febrile outpatients positive for Salmonella Typhi.[25]”* (lines 344-346) This point was discussed in the context of prescribing of Watch antibiotics.  There are no prior studies on antibiotic use in primary care in Zimbabwe for direct comparisons. |
| Line 266-268. The study's scale and duration are highlighted several times. Rather emphasize how this large dataset provides unique insights into prescribing trends and disease burden. | The sentence was removed as suggested to avoid repetitions. A point was made about the value of the data for prescribing and disease burden:  *“These data provide valuable insights into diagnoses and prescription across age groups which can be valuable for policy makers in this setting.”* (lines 445-446) |
| Line 273- 279. Explain diagnostic gaps causing overprescription and propose solutions such as point-of-care tests. | As suggested in the comment above, the discussion section was reorganised into themes. A statement was rephrased to specifically call for point-of-care tests that can reduce unnecessary prescriptions: *“These findings suggest that simple, inexpensive tests, ideally which can be done at the point-of-care, have the potential to reduce unnecessary prescriptions and optimise therapy in LMIC.”* (lines 369-371) |
| Line 277. The statement "only 19% of people in LMIC have access to diagnostics" is too broad and lacks specificity to Zimbabwe. | We fully agree that this statement is very broad and is not specific to Zimbabwe. Unfortunately exact data on the availability of diagnostics in the primary care setting are not available. Since this work has been submitted, another article was published which reported that 1% of laboratories in Zimbabwe have microbiology testing capacity. This also reflects the availability of services at lower levels of care and a reference was added in text: “*In Zimbabwe, access to microbiology services is also limited.” (doi: 10.1016/j.lanmic.2024.100976)* (line 360) |
| Line 282-285. There are high prescription rates for respiratory and diarrhoea which are not critically evaluated. Any systemic factors (e.g., diagnostic gaps, clinical guidelines) which drive these trends? How do they align with the guidelines. | From previous work done in the setting, we believe that diagnostic uncertainties play an important role in the decision to prescribe antibiotics. A sentence was added on diagnostic uncertainties and the lack of data and testing: *“The high rates of antibiotic prescribing are largely due to diagnostic uncertainties [26] and limited access to diagnostics resulting in scarce data on the local burden of infections and the prevalence of AMR.”* (lines 355-357)  While antibiotics are clearly overprescribed, the choice is largely in accordance with the national guidelines: *“While the choice of antibiotic was often in accordance with the WHO AWaRe Antibiotic Book and the national EDLIZ (Essential Medicines List and Standard Treatment Guidelines for Zimbabwe) recommendations[13, 20], antibiotics were likely unnecessary for the majority of episodes.[19]”* (lines 311-314) |
| Line 290-295. If not published why discussed it. Please describes clearly, it’s relevance to the study. | At the moment of writing this manuscript, we anticipated that the data would have been published. The sentence was removed in line with the editorial recommendations. |
| Line 305-314. Although the discussion mentions that most diarrheal episodes do not require antibiotics, it fails to evaluate how prescribing could be reduced. It will be interested to suggest interventions like treatment guidelines, Trainings programs etc. | Lines 355-379 discuss some of the interventions that can indirectly reduce unnecessary prescriptions including access to diagnostics, vaccination, access to safe water and sanitation. We have added a comment on training and access to the guidelines: *“Training initiatives on antibiotic prescribing may also play a role in improving antibiotic stewardship in Harare. In a study conducted in the same clinics from where the data were collected, health care workers reported that while they use the national guidelines to guide their practice, only a third had received training during the previous year on antibiotic prescribing.”* (lines 378-382)  Further explicit suggestions are also made in lines 431-433 :” …*additional, wider system interventions such as improving water and sanitation infrastructure, better governance, and increasing healthcare expenditures may be more impactful.[40, 41]”* |
| Line 315-331. The approach for managing STIs is described but not analyzed for its impact on overprescription or resistance. Discuss the risk in management, including overtreatment and missed diagnoses, and suggest alternatives like targeted diagnostic tools. | We added a paragraph on the burden of STIs in Zimbabwe, overtreatment and undertreatment including suggesting diagnostic testing. *“Sexually transmitted infections were particularly commonly diagnosed among younger adults where one out of six individuals received this diagnosis. Sexually transmitted infections are diagnosed and managed syndromically in Zimbabwe leading to both antibiotic overtreatment and undertreatment. A study conducted in Zimbabwe among individuals aged 16 to 24 years who underwent testing for Chlamydia trachomatis and Neisseria gonorrhoeae found that almost 20% of women and 10% of men had a positive test for sexually transmitted infections. However, only 40% of those reporting symptoms of sexually transmitted infections had a positive test, and most of those testing positive were asymptomatic.[22] These findings highlight the need for point-of-care testing for sexually transmitted infections in order to optimise antibiotic use and reduce transmission.”* (lines 323-332) |
| Line 329. The severe typhoid outbreaks during the study period are mentioned but not connected to prescribing practices or broader public health responses. Explain more on its impact. | A sentence on the impact of typhoid fever on antibiotic prescribing was added in the discussion: *“The difficulties in typhoid fever diagnosis can lead to considerable overprescribing with between three and 25 unnecessary antibiotic prescriptions for each true typhoid case, depending on the setting.”* (lines 316-318)  In response to the typhoid fever outbreak, Zimbabwe was the first country in Africa to conduct mass vaccination using the typhoid conjugate vaccine. Following vaccination, the number of typhoid cases decreased substantially. |
| Line 355- 361. Could you discuss how the double burden of NCDs and infectious diseases complicates antibiotic stewardship efforts. | A sentence was added on the impact on NCDs and older age on stewardship: *“Stewardship efforts might be challenged in these circumstances because of the presence of comorbidities influencing drug choice, polypharmacy, and the higher risk of infections and severe outcomes.”* (lines 400-402) |
| Line 366-367. The retrospective design is noted as a limitation, but potential biases in how healthcare workers diagnose and prescribe are not explored. | We agree that there may have been differences in practices among healthcare workers. A sentence on this issue was added to the limitations:  *“Diagnosis and management practices may have differed between healthcare workers and across clinics.”* (lines 413-414) |
| Line 368, if you mentioned the private sector, please explain clearly the underestimation on overall antibiotic use. | There are very few data in Zimbabwe on antibiotic prescriptions in the outpatient and community settings and for this reason we are unable to ascertain the degree of underestimation. While accessing private practices remains uncommon in Zimbabwe, access to antibiotics from private practices has likely increased.  Antibiotic access used to be highly regulated and in a study published in 2007, dispensing non-prescription antibiotics was uncommon in Zimbabwe (doi: 10.1093/jac/dkm013). The worsening economic situation has likely let to changes in antibiotic dispensing but there are no data on the extent to which people access antibiotics directly from private pharmacies (without a prescription). The lack of data for Zimbabwe is also shown in a recently published systematic review (<https://doi.org/10.1016/S1473-3099(23)00130-5>). |
| Line 370 372. The lack of data on dosage and duration is mentioned but not analyzed for its impact on the findings. Highlight how this affects the assessment of prescribing appropriateness. | We agree that the lack of dosages and durations impacts on our ability to evaluate whether prescriptions were appropriate for their target conditions. The lack of these data does not however impact on the main findings of our study which focused on diagnoses and whether and which antibiotics are prescribed in this setting.  We have added this as a limitation of our findings:  *“This also prevents an assessment of whether doses and durations of antibiotic treatment were appropriate and in accordance with the guidelines.”* (lines 419-421) |
| Conclusion  Line 390. Include a heading clearly for conclusion | A heading was added to the conclusion. |
| Lines 391–395: Focus on what your study is different, like the large dataset and its application to LMIC contexts. | The conclusion was rephrased as suggested to focus on the size of the dataset and the setting: *“This study, using a large dataset of routinely collected data from a low-resource settings, shows that infectious diseases…”* (lines 442-443) |
| Lines 391–395: Connect your results to other global issues such as AMR and provide solutions, for example stewardship programmes for Harare. | The section was revised to better highlight AMR and solutions: “*Excessive antibiotic prescribing accelerates the development and spread of AMR, rendering infections harder to treat. However, interventions that directly target prescribing may not be sufficient to reduce the AMR in LMIC and additional, wider system interventions such as improving water and sanitation infrastructure, better governance, and increasing healthcare expenditures may be more impactful”* (lines 429-433)  *“Our findings highlight the need for diagnostics in primary care and antibiotic stewardship in this setting to optimise patient management, reduce unnecessary antibiotic prescriptions, and curb the increase in AMR.”* (lines 446-448) |
| Limitations  Please clearly describe this in the study because it has been noted several times in different sections of the research. | The limitations have been reviewed in their specific section and throughout the discussion.  A heading was added to the section for clarity. |
| **REVIEWER 2** |  |
| This is a well written interesting paper, presenting an overview of antibiotic use in selected Harare health facilities, using routine clinical data. Such data are relatively rare to access in most of sSA, due to a lack of standardized digitally available surveillance data, but in view of increasing AMR and ABU (including Watch) highly relevant as a basis for public health policies and interventions to protect antibiotics and public health. | Thank you for your comments and your appreciation for our work. |
| Background  can data on prevalence of HIV and malnutrition be added | Data on the prevalence of HIV and malnutrition were added in the introduction:  “*Zimbabwe also has a high prevalence of HIV of 12.9% among adults [9] while 21.6% and 2.9% children under the age of 5 have either stunting or wasting, all of which increase the risk and severity of infections*.” (lines 110-112) |
| Methods  please clarify how the clinics were selected for inclusion, and to what extent this can have biased results? Eg access to care, socio-economic background, staffing, etc may vary by facility? | Clinics were selected based on their catchment population (“serving a high-density, low-income population”) and on the availability of medical records. All were public clinics under the same health authority and staffing organisation is expected to have been similar across clinics. While there were differences in socioeconomic backgrounds (within the low-income population), clinics included covered a range of incomes and occupations. We do not expect substantial bias due to the selection and thus results are generalisable to the low-income population of Harare.  A clarification was added:  *“Clinics were selected based on their catchment population and on the availability of medical records.”* (lines 119-120)  A sentence on the generalisability of these findings was added to the discussion: *“This study collected routine data from eight primary care clinics serving a population of more than 700,000 over seven years, making these findings generalisable for this setting.”* (lines 290-291) |
| can the authors clarify inclusion criteria? It is mentioned ‘acute illness’, but ‘acute’ is not defined. Eg it is a bit surprising this would include hypertension, while on the other hand it is likely to miss out on many other NCDs, impacting on the observations on the double burden of disease. Is there information on the total number of all consultations (acute and not acute)? | We included all patients seeking care for communicable and non-communicable diseases presenting to the outpatient clinic. We recognise that the term “acute” may be misleading in this context as clinic consultations also included patients with chronic conditions seeking care. For this reason we have changed the term of “acute presentations” to “outpatients”. The registers did not contain very specific information on whether a consultation was acute or chronic.  The study included patients with hypertension as well as with other NCDs. Hypertension was common in this dataset because it is a frequent condition which is more easily diagnosed at primary care level than other NCDs such as diabetes and chronic kidney disease. This is because blood pressure machines are sometimes available at the clinics.  The study excluded patients presenting to the TB and HIV clinics, patients presenting for antenatal care/ maternity and those presenting to the well-child clinic (growth monitoring and immunisations). These were all conducted in different departments of the clinic.  We have provided clarifications on the inclusion criteria:  *“Data on all recorded patient consultations from the outpatient department conducted between 1st January 2016 and 31st December 2022 were collected from clinic paper registries. Patients presenting to the tuberculosis and HIV clinics as well as those from the antenatal-, maternity and well-child clinics were excluded. “* (lines 135-138)  We have also replaced references to “acute presentations” throughout the manuscript. |
| -please clarify the referral process: it is mentioned that patients were ‘recommended to refer to hospital’: does this mean some may have gone, and others not; depending upon self-referral? Also,? it is mentioned that direct referrals were excluded from the analysis of antibiotics, but does this also relate to direct referrals who already received antibiotics? | This is correct. The nurses could only make a recommendation that the patient travels by him-/herself to the hospital. This means that some patients may have not been able/ willing to go to the hospital following a referral.  The referral process is described as follows  *“Patients with severe conditions are recommended to refer to hospital for further evaluation and care.”* (lines 127-128)  All referrals irrespective of whether they received antibiotics during the consultation or not were excluded from the antibiotic prescription analysis.  This was clarified in the methods section: *“…and all patients minus those who were directly referred to hospital or for whom the referral status was unknown for antibiotic prescriptions (irrespective of antibiotics receipt prior to referral).”* (lines 166-168) |
| -to what extent does the more generic category acute respiratory infection overlap with more specific categories for pneumonia and upper respiratory tract infections? Are these 3 categories used consistently over time, place and patient group? | Categories for pneumonia, upper respiratory tract infections and acute respiratory infections are mutually exclusive. Most of these diagnoses originate from direct coding from the medical records (selection of a drop-down option during data entry). A few overlaps were identified among manually-coded diagnoses which were corrected.  The category “respiratory tract infections” is meant to be broader and cover the sub-categories of respiratory tract infection diagnoses. To clarify this point a sentence was added to the method: *“Respiratory tract infections included the following diagnoses: upper respiratory tract infections (URTI), lower respiratory tract infections (LRTI), respiratory tract infections (RTI); acute respiratory infections (ARI), bronchitis, pneumonia, sinusitis, pharyngitis, tonsilitis, otitis, and COVID-19.”* (lines 146-149)  This categorisation is used consistently throughout the manuscript and analysis however the diagnoses are captured as recorded by the healthcare workers conducting the patient consultations. Therefore there may have been differences in practices across healthcare workers and clinics. This issue is acknowledged as a limitation *“Diagnosis and management practices may have differed between healthcare workers and across clinics.”* (lines 413-414) |
| -the diagnostic group ‘others’ is very large (a third to a quarter of all diagnosis), more details should be provided (the minimum would be a distinction into infectious/non-infectious). It is stated that it consists of diagnosis mentioned for less than 2%, but it might be possible to group some into larger categories (similar as presumably done for most of the specified categories, such as GE, STI, SSTI, trauma, ). Eg malaria and unclear fevers are rather common presentations in many sSA settings, which can trigger antibiotic use, and it would be relevant to show their contribution, and clarify to what extent malaria diagnostics were available/used. Also, this is not consistent throughout: in table 1 ‘rash’ is included as a separate category, in table 2 ‘urinary tract infections’; while in both tables ‘others’ is missing | Because this dataset contains very little clinical information and sometimes the diagnoses are relatively non-specific, we have refrained from making the categorisation into infectious and non-infectious conditions. While some diagnoses are clearly infectious (meningitis, pneumonia) or non-infectious (diabetes) for a considerable number this is more unclear (e.g. abdominal pain which might be due to typhoid fever or have non-infectious causes, headache, rash, etc.). To provide more information on the other diagnoses, we have added a table to the supplement showing further categories. (Table S2) Malaria is not endemic to Harare and therefore this diagnosis was uncommon (Table S2). For this reason, malaria is not expected to have substantially contributed to antibiotic prescriptions. Clinics usually have rapid malaria tests available to enable the diagnosis of those who travelled outside Harare.  For improved consistency, rash was removed from Table 1 and the other diagnosis category was added.  Table 2 is meant to reflect on quality indicators for antibiotic prescribing and this is the reason why urinary tract infections are included here (despite representing only 1.6% of diagnoses). |
| -data were captured from retrospective paper registers over a long period of time. Please provide some more information to what extent quality and completeness may have varied over time, and between centres | We acknowledge the fact that record data are incomplete and that there may have been differences in recording (and preserving records) between clinics. These data are recorded in paper registers and the space at the clinic does not always allow for secure archiving. In fact, we had visited a number of additional clinics from other areas from which medical registers could not be retrieved. This issue is acknowledged in the limitations section *“Our study is limited by its retrospective approach to data collection, the limited number of variables for which data were collected and incomplete recording often experienced in routine paper-based clinical registers.”* (lines 411-413)  To provide more clarity, we have also added a table (Table S1) to the supplement reporting on presentations according to year and clinic. |
| -please clarify the study period. It is stated data were collected between Jan 2020 and April 2022, capturing the period from Jan 2016 till December 2022; this does not seem possible | This was a requirement from the PLOS GPH editorial team to include both the period of data collection and the period when patients presented to the clinic. The section was amended to:  *“Data on all recorded patient consultations from the outpatient department conducted between 1st January 2016 and 31st December 2022 were collected from clinic paper registries. […] Data were collected retrospectively between 6th January 2020 and 16th April 2022 and included…”* (lines 135-139) |
| Results  -fever was not recorded for over a third of patients, but this may not have been random? Eg for patients presenting with hypertension or musculoskeletal pains, this may be less indicated; for presumed infectious diseases, this may be one of the indicators for antibiotic use. Can data be presented on antibiotic use of the main categories with and without fever? (in table 2 includes this for GE only) | We agree with the reviewer on this issue. Healthcare workers may have been more likely to measure temperature in those with symptoms of infection. We have added to the supplement a table (Table S3) reporting on antibiotic prescriptions among patients with a recorded temperature (febrile and non-febrile). |
| -to explore potential differences between men and women in antibiotic use, it would be useful to provide a sensitivity analysis which excludes children under the age of 10, as they represent about half of all patients, and could mask gender differences among adults | We have added as suggested a sensitivity analysis on antibiotic use for the major infectious diagnoses among patients of 10 years and above (Supplement Figure S6). This shows that prescriptions are largely similar for women and men for these syndromes. |
| -table 1: please include all diagnostic categories (figure 1 provides information on 11, not 8 categories); and clarify the category ‘rash’ as before it was stated this was part of ‘others’, in line with it comprising 1.8% of all diagnoses (‘others’ is one of the categories still to be included in the table, preferably with some more subdivision; see above). The text states that acute pharyngitis was the fourth most common diagnosis, which is captured as tonsillitis in table and figure, please be consistent. -check for consistency of the provided numbers. Eg it is stated several times that 199,980 consultations were recorded over the study period, but on page 9, 195,999 is given as the total (of which 29141 were referred (or recommended to refer?)) | We have initially not included the categories for non-infectious syndromes (trauma, musculoskeletal pain, hypertension). These were now added to Table 1. Rash was removed from the table for consistency across figures and tables. We have also added a table to the supplement describing in more detail other diagnoses. The nomenclature was changed from pharyngitis to tonsilitis for consistency.  Thank you for pointing out the discrepancy between 199,980 and 195,999. This is because for some patients, the referral status was unknown. It is likely that these patients were not referred as referrals would have been more likely to be documented in the records. As this cannot be known for certainty and to avoid further confusion, we added a new column to the Table 1 and rechecked and corrected the numbers throughout the manuscript. Those with an unknown referral status were also excluded from the antibiotics analyses. |
| Discussion  -it is remarkable that hardly any seasonality was observed; can the authors comment? | This analysis was not intended to focus on seasonality. We are currently undertaking a separate analysis focusing on seasonality and other climate-related variables (temperature, humidity, etc.) and specific syndromes. |
| Minor: -check spelling (eg in abstract: findings: .. patients not refereed to hospital .. | The error was corrected |
